# Appropriateness of Lumbar Spine Magnetic Resonance Imaging Requests: Guideline Concordance, Diagnostic Yield, Artificial-Intelligence Assessment and Derivation of a Simple Clinical Decision Rule

**DOI:** 10.3390/diagnostics16183028

**Published:** 2026-09-18

**Authors:** Ahmet Kürşat Kara, Veli Umut Turgut, Mehmet Ali Kızılay

**Affiliations:** Department of Neurosurgery, Antalya City Hospital, 07080 Antalya, Türkiye

**Keywords:** lumbar spine, magnetic resonance imaging, appropriateness, low back pain, clinical decision rule, artificial intelligence

## Abstract

**Background/Objectives:** Lumbar magnetic resonance imaging (MRI) is widely overused in low back pain. We assessed the appropriateness of lumbar MRI requests against three international guidelines and two independent large language model (LLM)-based artificial intelligence (AI) assessments, related appropriateness to diagnostic yield, and derived a simple decision rule to identify imaging likely to change management. **Methods:** In this single-centre retrospective study, 147 consecutive adults undergoing lumbar MRI for low back pain were analysed. Each request was classified using pre-imaging clinical variables according to operationalised ACR, NICE and ACP criteria and by two blinded LLM assessments (Claude, Anthropic; ChatGPT, OpenAI). The reference outcome was a management-changing MRI (subsequent surgery or interventional procedure); a four-item decision rule was derived by exhaustive search over clinical criteria optimising the Youden index. **Results:** Depending on the instrument, 57.8–72.1% of requests were inappropriate (ACR 66.0%; NICE 72.1%; ACP 66.0%; AI 57.8–64.6%). Agreement between instruments was substantial to near-perfect (κ 0.69–0.94; inter-AI κ 0.77). Red flags or neurological warning features were present in only 5.4%. MRI changed management in 33.3% overall—58.1–80.5% of appropriate versus 15.1–16.8% of inappropriate requests. A rule of four criteria—red flag or neurological warning feature, neurogenic claudication, purely radicular pain, or pre-imaging consideration of surgery/intervention—predicted management-changing MRI with 84% sensitivity and 81% specificity (Youden 0.64, versus 0.50–0.59 for the guidelines) and would have reduced imaging volume by 59.2%. **Conclusions:** Roughly two-thirds of lumbar MRI requests were guideline-inappropriate and rarely altered management. In this exploratory derivation cohort, a simple four-item rule showed promising predictive performance, but its apparent numerical advantage over existing guideline logic was not statistically confirmed and requires independent prospective validation; two blinded LLM assessments showed substantial agreement with guideline judgements, supporting further evaluation of LLM-based pre-order screening in independently validated settings.

## 1. Introduction

Low back pain is a leading cause of disability worldwide and one of the most frequent reasons for imaging referral [1]. Because the majority of episodes are self-limiting and serious underlying pathology is rare, all major guidelines—including those of the American College of Radiology (ACR), the United Kingdom National Institute for Health and Care Excellence (NICE) and the American College of Physicians (ACP)—recommend against routine early imaging in the absence of red flags or progressive neurological deficit [2,3,4,5,6].

Despite this consensus, inappropriate use of lumbar magnetic resonance imaging (MRI) remains highly prevalent, with systematic reviews estimating that roughly one-third of spinal imaging is inappropriate, and overuse rates in unselected settings reaching far higher [7,8]. Unnecessary imaging is not merely a financial burden: degenerative findings are near-ubiquitous in asymptomatic individuals and increase steeply with age [9,10], and their incidental detection promotes labelling, patient anxiety, further low-value care and even excess surgery, while early MRI in the absence of indications has been associated with worse occupational outcomes [11,12,13].

Two questions remain insufficiently answered in routine practice. First, how well do guideline-based appropriateness judgements identify the scans that actually matter—those that change patient management? Guidelines were designed primarily to limit harm, and their positive yield has been examined less often. Second, can the appropriateness assessment itself be automated? Large language models (LLMs) have recently shown promise in imaging decision support [14], and an automated pre-order screen based on structured clinical data could operate at the point of request without additional workload.

We therefore performed a retrospective appropriateness audit of consecutive lumbar MRI requests at a tertiary centre, with three aims: (1) to quantify the proportion of inappropriate requests according to operationalised ACR, NICE and ACP criteria and to measure the agreement of two independent LLM-based artificial intelligence (AI) assessments with these guidelines and with each other; (2) to relate appropriateness to diagnostic and management yield; and (3) to explore, in a data-driven manner, whether a simple clinical decision rule could identify management-changing imaging at least as accurately as existing guideline logic.

## 2. Materials and Methods

### 2.1. Study Design and Patients

This single-centre retrospective study was conducted in the Department of Neurosurgery of a tertiary referral hospital (Antalya City Hospital, Türkiye) and was approved by the Ethics Committee of Antalya City Hospital (approval no: 27/286; date of approval: 23 July 2026). Consecutive adult patients (≥18 years) who underwent lumbar spine MRI for low back pain with or without leg symptoms during a three-month period (April–July 2026) were reviewed. Of 149 consecutive requests with complete pre-imaging clinical data, two patients under 18 years of age were excluded, leaving 147 patients for analysis. Patients imaged for known malignancy staging, postoperative routine follow-up or non-degenerative indications outside the low back pain pathway were not included. All MRI requests included in the study originated from the neurosurgery outpatient clinic. However, in the local healthcare system, patients may directly access neurosurgical outpatient services for common symptoms such as low back or neck pain without prior referral from primary care or another specialty. Therefore, the cohort included a broad spectrum of symptom severity and was not limited to patients already selected as surgical candidates.

### 2.2. Data Collection

For each patient, a structured data-collection form was completed from the electronic medical record, comprising demographics; comorbidities (diabetes, hypertension, malignancy history, osteoporosis, immunosuppression); symptom duration (<6 weeks, 6–12 weeks, >12 weeks); pain pattern (axial, radicular, mixed axial–radicular, or neurogenic claudication); neurological findings (motor deficit, sensory loss, reflex change); red-flag indicators (suspicion of cauda equina syndrome, malignancy—weight loss or night pain, infection—fever, intravenous drug use or recent intervention, and significant trauma); inflammatory features; conservative treatment (≥6 weeks received or not, and response); whether surgery or an interventional procedure was being considered at the time of the request; prior lumbar MRI and prior lumbar surgery; the MRI finding; and the subsequent clinical management (unchanged, surgery, or injection/interventional procedure). Pre-imaging clinical variables were obtained from contemporaneous clinical documentation recorded at the initial assessment and at the time of the MRI request, before MRI results were available. The variable ‘surgery or interventional procedure under consideration’—referred to hereafter as ‘pre-imaging consideration of surgery/intervention’—represented the treating clinician’s judgement, based on the history and physical examination, that the patient might be a candidate for surgery or an interventional procedure if imaging demonstrated a concordant structural lesion; it did not represent a definitive or scheduled treatment decision. MRI findings and subsequent management were ascertained separately from subsequent clinical records and were not used to define this pre-imaging variable. All appropriateness classifications exclusively used pre-imaging information; MRI findings and subsequent management were used only as outcome variables.

### 2.3. Appropriateness Criteria

Guideline recommendations were operationalised as explicit rules applied uniformly to all patients. For operational purposes, a red-flag or neurological-warning-feature composite (referred to hereafter as ‘red flag’ for brevity) was defined as suspected cauda equina syndrome, suspected or known malignancy, suspected infection, significant trauma, immunosuppression, or motor deficit, in line with the red-flag constructs used in current guidelines [15,16]. Motor deficit was included as a neurological warning feature rather than being considered a conventional red flag per se, since guideline terminology more commonly emphasises progressive or severe neurological deficit. According to the ACR-based rule, imaging was appropriate in the presence of a red flag, when symptoms had persisted beyond six weeks despite at least six weeks of failed conservative treatment, or when surgery or an interventional procedure was under pre-imaging consideration [2]. According to the NICE-based rule, imaging was appropriate only in the presence of a red flag or when the result was likely to change management, operationalised as pre-imaging consideration of surgery or intervention [3]. According to the ACP-based rule, imaging was appropriate in the presence of a red flag, or in patients with radicular symptoms or neurogenic claudication unresponsive to at least six weeks of conservative treatment who were candidates for surgery or epidural intervention [4,5].

### 2.4. Artificial-Intelligence Assessment

In addition to the rule-based classifications, each request was independently assessed by two general-purpose large language models (AI-1: Claude, Anthropic, San Francisco, CA, USA; interface-displayed model label ‘claude-fable-5’; AI-2: ChatGPT, OpenAI, San Francisco, CA, USA; interface-displayed model label ‘GPT-5.6 Sol’). Both assessments were performed in August 2026 using the commercially available web interfaces. These labels are reported exactly as displayed by the respective interfaces at the time of assessment and should not be interpreted as independently archived or frozen API model identifiers. To assess run-to-run reproducibility, each model was additionally re-run twice in September 2026, approximately one month after the primary assessment, using the identical prompt, dataset and web interface in new, independent chat sessions; agreement between runs was quantified with percentage agreement and Cohen’s κ, and the primary (August 2026) ratings were retained for all main analyses (Supplementary Table S3). Each model was provided with the identical structured pre-imaging clinical variables of each patient—demographics, comorbidities, symptom duration and pattern, neurological findings, red-flag indicators, conservative treatment history and interventional plans—and asked to judge whether lumbar MRI was appropriate or inappropriate. Both models were blinded to the MRI findings and to subsequent management, their holistic judgements were not constrained to any single guideline, and neither model had access to the other’s assessments. Each model received a clean dataset containing exclusively the pre-imaging clinical variables—without MRI findings, subsequent management or the other model’s judgements. The standardised prompt instructed each model to assess, for each patient independently and based only on the clinical information provided, whether lumbar spine MRI was ‘Appropriate’ or ‘Inappropriate’, to choose exactly one of these two categories, and to return only a two-column table of patient identifier and assessment; no free-text rationale was requested. The models were also explicitly instructed not to infer missing information. Because the models were supplied exclusively with pre-imaging variables, MRI findings, subsequent treatment and clinical outcomes were unavailable to them by construction. The complete verbatim prompt and the exact set of variables supplied to the models are provided in the Supplementary Materials (Supplementary Text S1). The large language models named above (Claude and ChatGPT) were used exclusively as the object of investigation—that is, as automated raters whose appropriateness judgements were evaluated—and not as tools for producing the manuscript; no generative artificial intelligence was used for the study design, the statistical analysis or the interpretation of the results. Separately, a generative AI tool was used solely to assist with language editing during manuscript preparation, as disclosed in the Acknowledgments.

### 2.5. Outcomes

The primary descriptive outcome was the proportion of inappropriate MRI requests according to each instrument. Diagnostic yield was described by the MRI finding (normal, degenerative change, disc herniation, spinal stenosis, compression fracture, or other). The reference outcome for predictive analyses was a management-changing MRI, operationally defined as MRI followed by surgery or an interventional procedure for the presenting lumbar condition; imaging followed by unchanged conservative management was considered non-contributory. This pragmatic endpoint was intended to capture downstream management escalation and should not be interpreted as a gold standard for imaging appropriateness or patient benefit, because an appropriate MRI may also support continued conservative management without leading to a procedure.

### 2.6. Derivation of the Decision Rule

Candidate pre-imaging predictors of a management-changing MRI were screened univariately. A multivariable logistic regression model and a shallow decision tree, each evaluated with stratified five-fold cross-validation, served as performance benchmarks. An interpretable decision rule was then derived by exhaustive enumeration of all combinations of two to five binary clinical criteria, classifying imaging as indicated when any criterion was met, and selecting the combination that maximised the Youden index. For the purposes of the derived rule, neurogenic claudication and purely radicular pain were treated as distinct symptom categories and were not included within the operational red-flag composite defined in Section 2.3. The superiority of the selected rule over the best-performing guideline was examined with a bootstrap confidence interval (5000 resamples) for the difference in Youden index. Because the criterion of pre-imaging consideration of surgery/intervention is closely related to the reference outcome, a sensitivity analysis was performed in which this criterion was removed from the derived rule and, where applicable, from the operationalised ACR and NICE algorithms, and the performance of the remaining purely clinical criteria was evaluated. Because the rule was derived and evaluated in the same cohort, its performance estimates should be regarded as optimistic pending external validation.

### 2.7. Statistical Analysis

Categorical variables are presented as a number (percentage) with Wilson 95% confidence intervals (CI) for key proportions, and continuous variables as mean ± standard deviation. Group comparisons used the Fisher exact test and the Mann–Whitney U test. Agreement between appropriateness instruments was quantified with Cohen’s kappa (κ). Because κ is sensitive to imbalanced marginal distributions, the prevalence-adjusted bias-adjusted kappa (PABAK) was also calculated for each pair of instruments. Predictive performance was described by sensitivity, specificity, positive and negative predictive values, accuracy and the Youden index. A two-sided *p* < 0.05 was considered significant. Analyses were performed in Python 3.11 (Python Software Foundation, Wilmington, DE, USA) using the SciPy (version 1.17), scikit-learn (version 1.8) and statsmodels (https://www.statsmodels.org) packages. The derivation and internal assessment of the decision rule are reported in accordance with the TRIPOD statement (Supplementary Table S4).

## 3. Results

### 3.1. Cohort Characteristics

A total of 147 patients (74 female, 73 male; mean age 47.8 ± 16.1 years, range 18–84) were analysed (Table 1). Symptom duration was under six weeks in 68 patients (46.3%), 6–12 weeks in 19 (12.9%) and over 12 weeks in 60 (40.8%). Pain was purely axial in 60 patients (40.8%), mixed axial–radicular in 50 (34.0%), purely radicular in 30 (20.4%) and neurogenic claudication in 7 (4.8%). At least one red flag or neurological warning feature was present in only eight patients (5.4%, 95% CI 2.8–10.4): a stable motor deficit in four and a history of malignancy in four. Fifty patients (34.0%) had received at least six weeks of conservative treatment. In the whole cohort, 56 (38.1%) had responded to conservative treatment, 41 (27.9%) had not, and in 50 (34.0%) no adequate conservative treatment was given before imaging. Surgery or an interventional procedure was under consideration at the time of request in 36 patients (24.5%).

### 3.2. Appropriateness of Requests

Depending on the instrument, 57.8% to 72.1% of lumbar MRI requests were classified as inappropriate: 97/147 (66.0%, 95% CI 58.0–73.2) by the ACR-based rule, 106/147 (72.1%, 95% CI 64.4–78.7) by the NICE-based rule, 97/147 (66.0%) by the ACP-based rule, 95/147 (64.6%, 95% CI 56.6–71.9) by AI-1 and 85/147 (57.8%, 95% CI 49.7–65.5) by AI-2 (Table 2). Ninety-five requests (64.6%) were inappropriate according to all three guidelines simultaneously, and 41 (27.9%) were appropriate according to all three. Although the ACR- and ACP-based rules each classified 97 requests as inappropriate, their patient-level classifications were not identical: two requests were appropriate by ACR but inappropriate by ACP, and two the reverse (κ = 0.94). Agreement between the guideline rules was high (κ 0.86–0.94), and both AI assessments substantially agreed with the guidelines (AI-1: κ 0.77–0.91; AI-2: κ 0.69–0.77) and with each other (κ 0.77). The 16 inter-model disagreements (10.9%) were concentrated in chronic axial-predominant pain—particularly in older patients—and in neurogenic claudication, both of which AI-2 tended to judge as appropriate. PABAK values were close to the corresponding κ values (guideline pairs 0.88–0.95; AI-1 versus guidelines 0.80–0.92; AI-2 versus guidelines 0.71–0.78; between models 0.78), indicating that agreement was not inflated by imbalanced marginal distributions. Run-to-run reproducibility was high for both models: across three runs performed one month apart, agreement between runs was 91.8–94.6% for AI-1 (κ 0.82–0.88; identical decisions in 89.1% of patients across all three runs) and 91.2–93.9% for AI-2 (κ 0.82–0.87; identical decisions in 88.4%), and agreement with the guideline rules and predictive performance were stable across runs (Supplementary Table S3). The most frequent patterns underlying inappropriate requests were imaging despite a documented response to conservative treatment (47 patients), acute symptoms of less than six weeks without red flags and without a trial of conservative treatment (30 patients), and chronic symptoms without an adequate conservative treatment trial (16 patients).

### 3.3. Diagnostic and Management Yield

**Figure 1:**
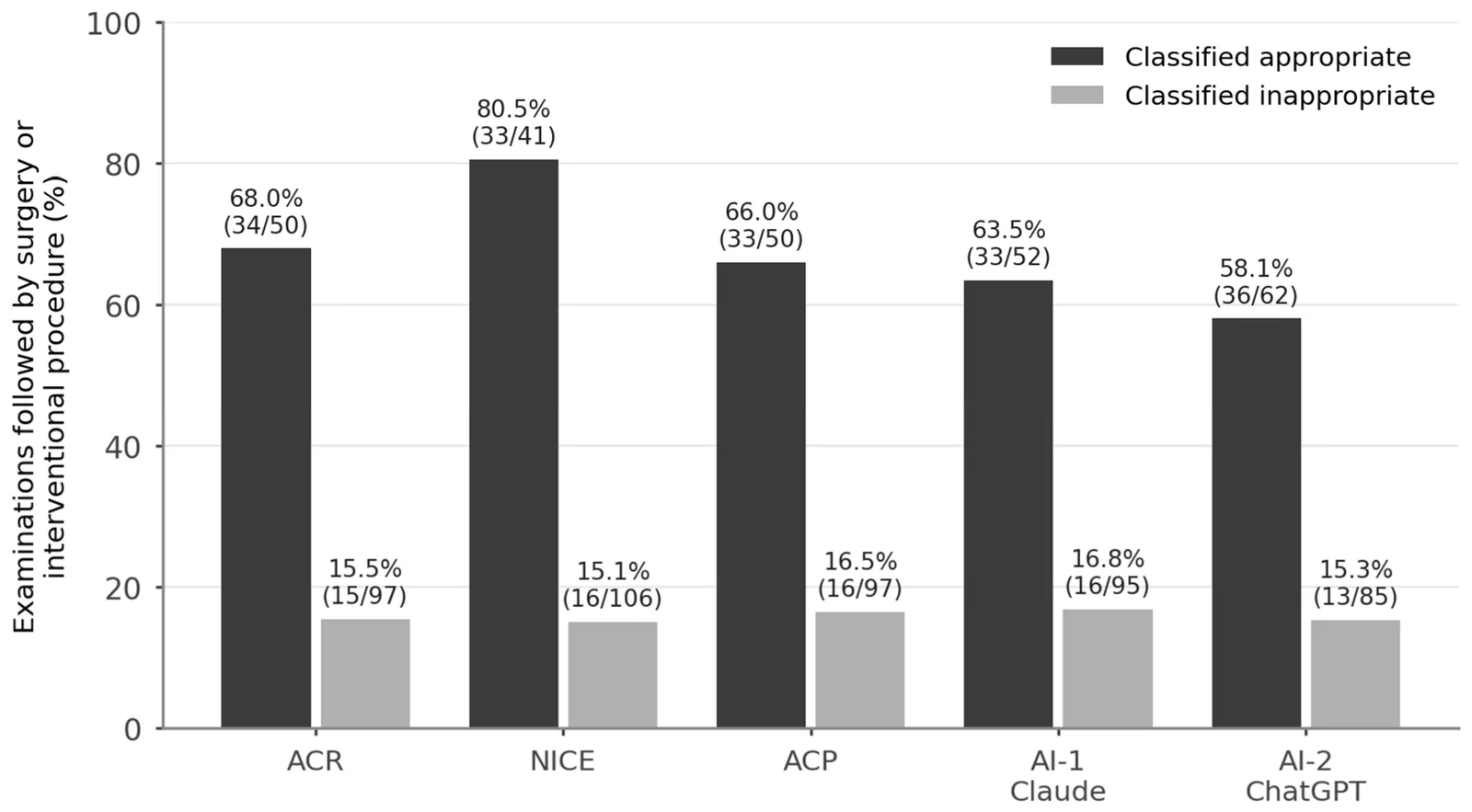
Management-changing yield according to MRI appropriateness classification. For each guideline-based rule and LLM assessment, the figure shows the proportion of examinations followed by surgery or an interventional procedure among requests classified as appropriate and inappropriate. Management-changing yield was consistently higher among appropriate examinations across all five instruments. ACR: American College of Radiology; NICE: National Institute for Health and Care Excellence; ACP: American College of Physicians; AI-1: Claude (Anthropic); AI-2: ChatGPT (OpenAI); LLM: large language model.

MRI demonstrated only degenerative change in 50 patients (34.0%), disc herniation in 39 (26.5%), a normal study in 33 (22.4%), spinal stenosis in 20 (13.6%), compression fracture in 4 (2.7%), and a sacral perineural (Tarlov) cyst in 1 patient (0.7%); the latter was an incidental finding and did not result in a change in management. Management changed after imaging in 49 patients (33.3%): surgery in 37 and an interventional procedure in 12. Yield was strongly concentrated in appropriate requests: across the five instruments, management changed after 58.1–80.5% of scans classified as appropriate, compared with only 15.1–16.8% of scans classified as inappropriate, depending on the instrument (Table 3; Figure 1). Among ACR-inappropriate requests, 67.0% (65/97) showed a normal or purely degenerative study and 84.5% (82/97) led to no change in management (i.e., management changed in 15/97, 15.5%), whereas management changed after 68.0% (34/50) of ACR-appropriate requests (*p* < 0.001).

### 3.4. Predictors of a Management-Changing MRI and the Derived Rule

In univariate analysis, the strongest pre-imaging predictors of a management-changing MRI were pre-imaging consideration of surgery or intervention (odds ratio [OR] 24.2, *p* < 0.001), neurogenic claudication (management changed in 7/7 patients, *p* < 0.001), motor deficit (4/4, *p* = 0.011), purely radicular pain (OR 3.5, *p* = 0.004), failed conservative treatment (OR 3.4, *p* = 0.002), symptom duration over 12 weeks (OR 3.1, *p* = 0.002), red flag or neurological warning feature (OR 6.7, *p* = 0.017) and older age (mean 55.1 vs. 44.1 years, *p* < 0.001), whereas a documented response to conservative treatment predicted a non-contributory scan (OR 0.5, *p* = 0.048). A cross-validated logistic regression using these variables achieved an area under the curve of 0.86 (stratified five-fold cross-validation; apparent in-sample performance is not reported).

**Figure 2:**
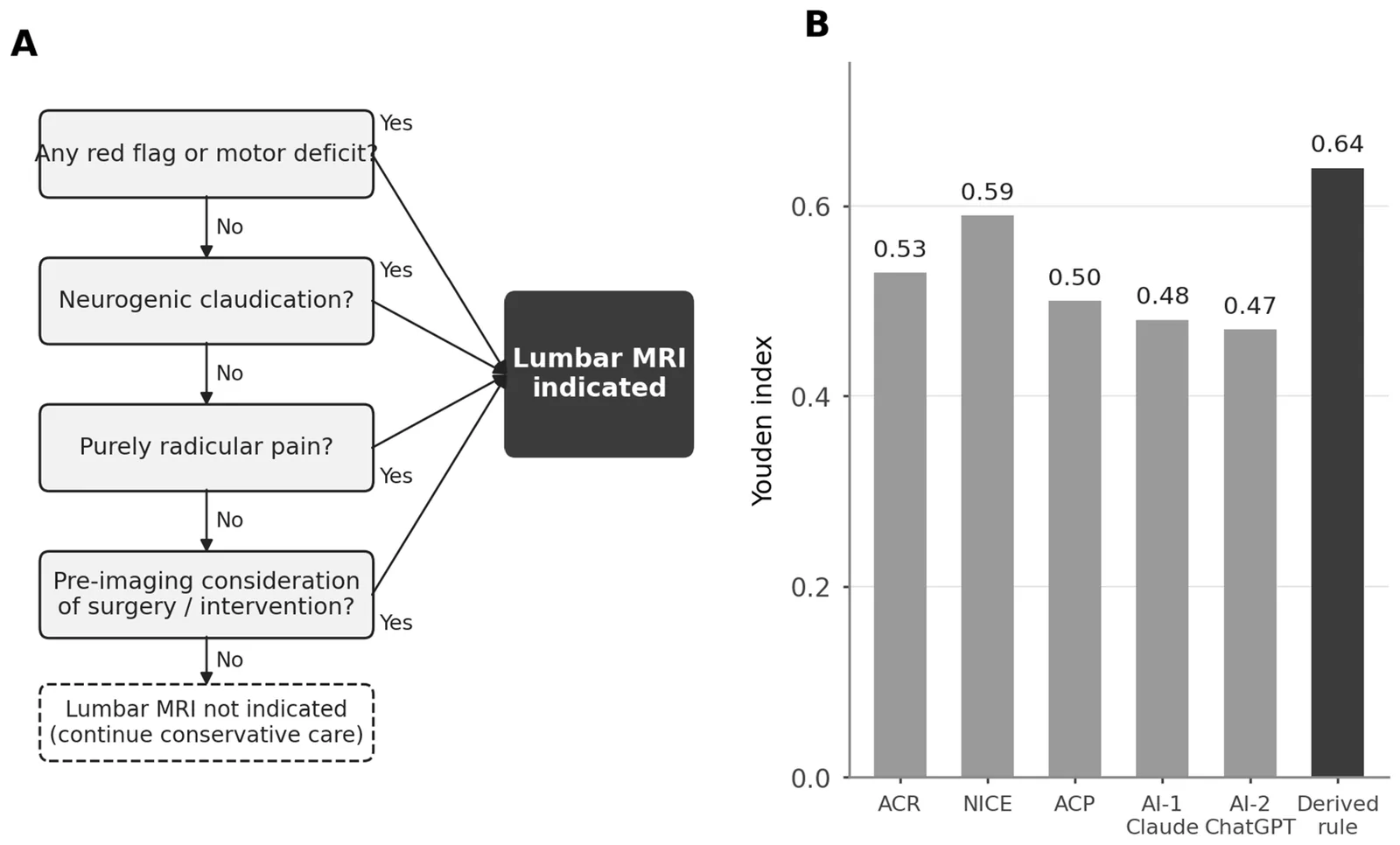
Visual summary of the derived decision rule and comparative predictive performance. (**A**) Flowchart of the four-item rule indicating lumbar MRI when at least one of the following is present: red flag or neurological warning feature (motor deficit), neurogenic claudication, purely radicular pain, or pre-imaging consideration of surgery/intervention. (**B**) Comparison of Youden indices across the operationalised ACR, NICE and ACP rules, the two LLM assessments, and the derived four-item rule. ACR: American College of Radiology; NICE: National Institute for Health and Care Excellence; ACP: American College of Physicians; AI-1: Claude (Anthropic); AI-2: ChatGPT (OpenAI); LLM: large language model.

The exhaustive rule search selected a four-item rule (Figure 2A): lumbar MRI is indicated when at least one of the following is present—(1) any red flag or neurological warning feature; (2) neurogenic claudication; (3) purely radicular pain; or (4) pre-imaging consideration of surgery or intervention. This rule identified management-changing imaging with a sensitivity of 84% (41/49; 95% CI 71–91%), specificity of 81% (79/98; 95% CI 72–87%), positive predictive value of 68% (95% CI 56–79%), negative predictive value of 91% (95% CI 83–95%) and accuracy of 82% (95% CI 75–87%) (Youden index 0.64, bootstrap 95% CI 0.51–0.77), compared with Youden indices of 0.53 for ACR, 0.59 for NICE and 0.50 for ACP (Table 4; Figure 2B). If applied to this cohort, the rule would have avoided 87 of 147 examinations (59.2%) while retaining 84% of the management-changing scans. The bootstrap difference in Youden index between the rule and the best-performing guideline (NICE) was +0.05 (95% CI −0.07 to +0.17), indicating a favourable but not yet statistically confirmed advantage at this sample size. Of the eight management-changing scans missed by the rule, seven were in patients with axial-predominant pain in whom an injection procedure was performed. These seven patients (six with purely axial and one with mixed axial–radicular pain) had a mean age of 45.9 ± 12.8 years (range 24–60); five were female. Symptom duration exceeded 12 weeks in four, was under 6 weeks in two and was 6–12 weeks in one. Only two of the seven had received at least six weeks of conservative treatment, whereas four had a documented response to conservative treatment before imaging; none had a neurological deficit or red flag. MRI showed degenerative change only in four, disc herniation in two and a normal study in one, and six of the seven requests were classified as inappropriate by all five instruments. Their individual characteristics are provided in Supplementary Table S2. In a sensitivity analysis addressing the potential influence of pre-imaging consideration of surgery or intervention, this criterion was removed from the derived rule and from the rule-based guideline algorithms where applicable. The resulting three-item rule, based on red flags, neurogenic claudication and purely radicular pain, had a sensitivity of 0.55, a specificity of 0.85 and a Youden index of 0.40. Removal of the analogous criterion also reduced the Youden index of the operationalised ACR rule from 0.53 to 0.31 and of the NICE rule from 0.59 to 0.10 (Supplementary Table S1).

## 4. Discussion

In this consecutive series of lumbar MRI requests at a tertiary neurosurgical centre, roughly two out of three examinations were considered inappropriate by operationalised ACR, NICE and ACP criteria, red flags or neurological warning features were present in only one in twenty patients, and the majority of inappropriate scans showed normal or purely degenerative findings and altered management in roughly one in six cases or fewer. These figures exceed the approximately one-third pooled inappropriateness reported in systematic reviews of spinal imaging [7,8], although rates vary widely with setting and methodology, and they translate the abstract notion of ‘low-value imaging’ into a concrete local figure: had imaging been restricted to guideline-concordant requests, more than half of the examinations—along with their cost, scanner time and waiting-list burden—would have been avoided at the price of missing a minority of management-changing findings.

The anatomy of inappropriateness in our cohort is instructive, because the dominant patterns are organisational rather than diagnostic dilemmas. The single largest group comprised patients imaged despite a documented response to conservative treatment, followed by patients imaged within six weeks of symptom onset without red flags and without any trial of conservative therapy. Both patterns are explicitly discouraged by all three guidelines [2,3,4,5,6] and are unlikely to reflect genuine clinical uncertainty; they are more plausibly driven by patient expectation, time pressure and the perceived reassurance value of imaging—mechanisms well described in the overuse literature [8,12]. The harms are equally well documented: degenerative findings are common in asymptomatic individuals [9,10], and early or unindicated MRI has been associated with increased subsequent healthcare utilisation, disability and surgery without corresponding benefit [11,12,13,17].

The second contribution of this study is the explicit confrontation of appropriateness with yield. Guideline-appropriate requests were followed by a change in management in about two-thirds to four-fifths of cases, whereas inappropriate requests changed management in approximately 15–17%—a four- to five-fold difference that held across all instruments. This supports the internal validity of guideline logic, but also exposes its ceiling: about one-third of guideline-appropriate scans still altered nothing, and a small number of guideline-inappropriate scans preceded interventions, chiefly injections in axial-predominant pain. The value of pre-injection MRI in axial pain is itself debatable, and this subgroup accounted for most of the residual disagreement between guideline judgement and observed management. Given the limited number of guideline-inappropriate examinations that nevertheless preceded a management-changing decision, this subgroup should be considered exploratory and warrants targeted evaluation in a larger prospective cohort.

Building on these observations, a data-driven search across all combinations of pre-imaging criteria converged on a compact four-item rule—red flag or neurological warning feature, neurogenic claudication, purely radicular pain, or pre-imaging consideration of surgery/intervention—that showed a numerically better predictive performance than all three guidelines in Youden terms (0.64 versus 0.50–0.59) in this derivation cohort, with a sensitivity of 84% and a 59.2% reduction in the number of scans. The rule is clinically coherent: it unites the safety net of red flags and neurological warning features with the three presentations in which imaging most plausibly informs an actionable decision. Two caveats must be stated plainly. First, the rule was derived and evaluated in the same cohort of 147 patients; its apparent advantage over NICE did not reach statistical confirmation (bootstrap 95% CI for the Youden difference −0.07 to +0.17), and derivation-set performance is systematically optimistic [18]. Second, although the criterion of pre-imaging consideration of surgery/intervention is temporally and conceptually distinct from the outcome—it reflects the clinician’s pre-imaging judgement, not an already scheduled procedure—the two are closely related, since patients judged to be candidates for intervention are more likely to proceed to it. This predictor–outcome proximity may inflate the apparent performance of the rule, although a closely related construct underlies the NICE recommendation that imaging be reserved for situations in which the result is likely to change management [3]. When pre-imaging interventional consideration was removed, the performance of the derived rule decreased, confirming that this variable materially contributed to its discrimination. However, a similar or greater reduction was observed when the analogous criterion was removed from the operationalised ACR and NICE algorithms. This finding suggests that the concept of pre-imaging interventional candidacy is not unique to the derived rule but is embedded, as an analogous clinical construct, in the logic of both the ACR and NICE recommendations—although NICE expresses it more broadly, as imaging being appropriate when the result is expected to change management, and our operationalisation of that criterion as pre-imaging consideration of surgery or intervention is necessarily narrower. Nevertheless, the lower sensitivity of the three-item rule reinforces the need for independent prospective validation. The principal failure mode of the rule involved patients with axial-predominant pain who subsequently underwent injection procedures; in most of these, MRI showed only non-specific degenerative change or was normal, and several had already responded to conservative treatment. This subgroup may represent a clinically distinct population in whom imaging decisions are influenced by procedural planning not fully captured by conventional radicular or claudicant symptom patterns. Given its small size, we do not propose modifying the rule on this basis; the subgroup rather identifies an area requiring further prospective study. Because the definition of red flags and warning neurological features varies across guidelines, the performance of the derived rule may depend partly on the operational definitions used in the present study and should be reassessed using alternative guideline-specific definitions in future validation cohorts. The rule should therefore be regarded as hypothesis-generating and requires validation in an independent, ideally prospective cohort, which is planned as the present registry expands.

Our findings extend the emerging literature on LLM-based imaging appropriateness assessment by evaluating two independent general-purpose LLMs against three guideline-based classifications in the same lumbar MRI cohort: the substantial-to-near-perfect agreement of both models with all three guidelines (AI-1: κ 0.77–0.91; AI-2: κ 0.69–0.77) and with each other (κ 0.77) indicates that general-purpose language models supplied only with structured pre-imaging variables can reproduce multi-guideline appropriateness judgements in spinal imaging. Notably, the two models diverged in only 16 cases, and AI-2 achieved a somewhat higher sensitivity for management-changing imaging (0.73 versus 0.67–0.69 for the guidelines) at the cost of lower specificity, largely by judging neurogenic claudication appropriate—the same presentation identified independently by the data-driven rule. Recent work has reported encouraging performance of LLMs in radiologic decision support [14], and subsequent studies have extended these observations to musculoskeletal and spine imaging, demonstrating promising LLM performance in assessing musculoskeletal MRI appropriateness against ACR criteria [19], in enhancing and protocoling spine MRI request forms with high run-to-run reproducibility [20], and in vetting outpatient lumbar spine MRI referrals [21]. The practical implication is not that AI should adjudicate imaging, but that an automated pre-order screen could flag probably inappropriate requests in real time at negligible marginal cost, reserving clinician review for flagged cases—a strategy compatible with clinical-decision-support mandates for advanced imaging.

The potential implementation of LLM-based appropriateness screening also raises questions regarding generalisability and algorithmic bias. Model performance may vary according to language, healthcare-system characteristics, referral patterns and the populations represented in model training data. The present study was conducted in a Turkish tertiary-care setting, and the observed agreement with guideline-based assessments should therefore not be assumed to generalise directly to other countries or healthcare environments. Before clinical implementation, LLM-based screening tools should be prospectively evaluated across diverse populations, languages, specialties and care settings.

This study has limitations. It is retrospective and single-centre. Although the sample of 147 patients was sufficient to estimate the proportion of inappropriate imaging with approximately ±8% precision, it was relatively small for the derivation of a multivariable clinical decision rule. Accordingly, the observed performance of the four-item rule should be interpreted as hypothesis-generating rather than confirmatory. The number of management-changing events (*n* = 49) was also modest relative to the number of candidate predictors, which may have limited the stability of the multivariable logistic-regression estimates despite cross-validation. The bootstrap confidence interval for the difference in Youden index between the derived rule and the best-performing guideline crossed zero, indicating that superiority over existing guideline logic was not statistically established. A future prospective validation study should therefore use an independently recruited cohort and a prespecified sample-size calculation based on the expected prevalence of management-changing MRI examinations and the desired precision of sensitivity and specificity estimates. The reference outcome—a change in management—is a pragmatic but imperfect surrogate for patient benefit: an appropriate scan may correctly lead to continued conservative care, and an intervention does not guarantee improvement. Because the outcome was defined pragmatically by subsequent management, it does not imply that the MRI finding itself causally determined the intervention in every case. The guideline recommendations were operationalised as binary appropriate/inappropriate rules to permit direct patient-level comparison across instruments. This simplification does not fully preserve the scenario-based and graded nature of the original recommendations, particularly for ACR, and may therefore have shifted the observed proportion of inappropriate examinations in either direction. Our findings should consequently be interpreted as reflecting the performance of the operationalised guideline algorithms used in this study rather than the full nuance of the source guideline documents. Although all requests originated from a neurosurgery outpatient clinic, direct patient access to neurosurgical services in our healthcare system means that the study population was not restricted to highly selected surgical referrals. Nevertheless, referral pathways and the pre-test probability of structural disease differ between settings, and the observed rates of MRI inappropriateness, diagnostic yield and management change may therefore not be directly generalisable to primary care, emergency, rheumatology, orthopaedic or other outpatient populations. Data on prior imaging were incomplete and were not used in the models. Finally, the AI assessment was performed by two independent models with substantial inter-model agreement, and run-to-run reproducibility was high when the identical prompt was re-applied one month later (Supplementary Table S3); however, the sensitivity of LLM judgements to alternative prompt formulations and their stability across future model versions were not examined and remain important prerequisites for clinical deployment.

## 5. Conclusions

Approximately two-thirds of lumbar MRI requests in this cohort were inappropriate by ACR, NICE and ACP criteria, and such scans rarely changed management. The derived four-item rule—red flag or neurological warning feature, neurogenic claudication, purely radicular pain, or pre-imaging consideration of surgery/intervention—showed promising discrimination for management-changing imaging in this exploratory cohort and would have reduced imaging volume by 59.2%; however, its performance estimates are likely optimistic and require independent prospective validation. Two independent LLM-based assessments reproduced guideline judgements with substantial-to-near-perfect agreement. Pending prospective validation, these findings support further evaluation of the structured, potentially automated screening of lumbar MRI requests at the point of order.

## Figures and Tables

**Table 1:** Baseline and clinical characteristics of the cohort (*n* = 147).

Characteristic	*n* (%) or Mean ± SD
Age, years	47.8 ± 16.1
Female sex	74 (50.3)
Symptom duration <6 weeks/6–12 weeks/>12 weeks	68 (46.3)/19 (12.9)/60 (40.8)
Pain pattern: axial/mixed/radicular/neurogenic claudication	60 (40.8)/50 (34.0)/30 (20.4)/7 (4.8)
Any red flag or neurological warning feature	8 (5.4)
Motor deficit	4 (2.7)
History of malignancy	4 (2.7)
≥6 weeks of conservative treatment received	50 (34.0)
Response to conservative treatment: responded/failed/not given	56 (38.1)/41 (27.9)/50 (34.0)
Pre-imaging consideration of surgery or intervention	36 (24.5)

**Table 2:** Appropriateness of lumbar MRI requests by instrument, and agreement between instruments.

Instrument	Inappropriate, *n* (%)	95% CI	κ vs. ACR	κ vs. NICE	κ vs. ACP	κ vs. AI-1
ACR-based rule	97 (66.0)	58.0–73.2	–	0.86	0.94	0.91
NICE-based rule	106 (72.1)	64.4–78.7	0.86	–	0.86	0.77
ACP-based rule	97 (66.0)	58.0–73.2	0.94	0.86	–	0.85
AI-1 (Claude)	95 (64.6)	56.6–71.9	0.91	0.77	0.85	–
AI-2 (ChatGPT)	85 (57.8)	49.7–65.5	0.77	0.69	0.77	0.77

Inappropriate by all three guidelines simultaneously: 95/147 (64.6%). ACR: American College of Radiology; NICE: National Institute for Health and Care Excellence; ACP: American College of Physicians; AI-1: Claude (Anthropic); AI-2: ChatGPT (OpenAI); CI: confidence interval (Wilson); κ: Cohen’s kappa. Prevalence-adjusted bias-adjusted kappa (PABAK): guideline pairs 0.88–0.95; AI-1 versus guidelines 0.80–0.92; AI-2 versus guidelines 0.71–0.78; AI-1 versus AI-2 0.78.

**Table 3:** MRI findings and subsequent management according to ACR-based appropriateness.

	Appropriate (*n* = 50)	Inappropriate (*n* = 97)
Normal or purely degenerative MRI, *n* (%)	18 (36.0)	65 (67.0)
Disc herniation/stenosis/fracture/other, *n* (%)	32 (64.0)	32 (33.0)
Management unchanged, *n* (%)	16 (32.0)	82 (84.5)
Surgery, *n* (%)	29 (58.0)	8 (8.2)
Injection/interventional procedure, *n* (%)	5 (10.0)	7 (7.2)

Management changed after 68.0% of appropriate versus 15.5% of inappropriate requests (*p* < 0.001, Fisher exact test).

**Table 4:** Performance of appropriateness instruments and of the derived four-item rule in predicting a management-changing MRI.

Instrument	Sensitivity	Specificity	PPV	NPV	Accuracy	Youden index	Scans Performed, *n*
ACR-based rule	0.69	0.84	0.68	0.85	0.79	0.53	50
NICE-based rule	0.67	0.92	0.80	0.85	0.84	0.59	41
ACP-based rule	0.67	0.83	0.66	0.84	0.78	0.50	50
AI-1 (Claude)	0.67	0.81	0.63	0.83	0.76	0.48	52
AI-2 (ChatGPT)	0.73	0.73	0.58	0.85	0.73	0.47	62
Derived four-item rule	0.84 (0.71–0.91)	0.81 (0.72–0.87)	0.68 (0.56–0.79)	0.91 (0.83–0.95)	0.82 (0.75–0.87)	0.64 (0.51–0.77)	60

Derived rule: lumbar MRI is indicated if any of the following are present: red flag or neurological warning feature; neurogenic claudication; purely radicular pain; pre-imaging consideration of surgery/intervention. PPV/NPV: positive/negative predictive value. For the derived rule, 95% confidence intervals are given in parentheses (Wilson method; bootstrap for the Youden index). Reference outcome: MRI followed by surgery or an interventional procedure (49/147, 33.3%).

## Data Availability

The anonymised dataset supporting the findings of this study is available from the corresponding author on reasonable request. The standardised prompt and the list of variables supplied to the language models are presented in the Supplementary Materials (Supplementary Text S1).

## Supplementary Materials

The following supporting information can be downloaded at: https://www.mdpi.com/article/10.3390/diagnostics16183028/s1, Supplementary Text S1: Standardised prompt and list of pre-imaging variables supplied to the large language models; Table S1: Sensitivity analysis excluding pre-imaging consideration of surgery or intervention from the rule-based instruments and from the derived rule; Table S2: Individual characteristics of the seven patients with axial-predominant pain and a subsequent injection procedure whose management-changing MRI was not identified by the four-item rule; Table S3: Run-to-run reproducibility of the large language model assessments; Table S4: Completed TRIPOD checklist for the derivation and internal assessment of the clinical decision rule.
