# Supplementary material for: Appropriateness of Lumbar Spine Magnetic Resonance Imaging Requests: Guideline Concordance, Diagnostic Yield, Artificial-Intelligence Assessment and Derivation of a Simple Clinical Decision Rule
Source: Diagnostics (Basel). 2026 Sep 18;16(18):3028. doi: 10.3390/diagnostics16183028 (PMC13605444; doi:10.3390/diagnostics16183028)
Supplement: Supplementary file 1 [file diagnostics-16-03028-s001.zip › diagnostics-4534384-supplementary.pdf]

Supplementary Materials

Supplementary Text S1. Standardised prompt and list of pre-imaging variables supplied to the large language models.

Verbatim prompt (identical for both models and for all runs):

> You are given a table containing pre-imaging clinical information for patients.
>
> For each patient, independently assess whether lumbar spine MRI is Appropriate or Inappropriate based only on the clinical information provided.
>
> Do not infer missing information.
>
> For every patient, choose exactly one of the following: Appropriate / Inappropriate.
>
> Return only a two-column table: Patient ID | Assessment

Variables supplied to the models (one row per request; MRI findings, subsequent management and outcome data were not included):

Variable (column name as supplied)	Permitted values
Request_ID	Coded identifier (R001–R147)
Age_years	Years
Sex	Female / Male
Diabetes	Yes / No
Hypertension	Yes / No
History_of_malignancy	Yes / No
Osteoporosis	Yes / No
Immunosuppression_or_chronic_steroid	Yes / No
Other_comorbidity	Free text (e.g., hyperlipidaemia) or blank
Symptom_duration	<6 weeks / 6–12 weeks / >12 weeks
Pain_pattern	Axial only / Mixed axial and radicular / Radicular only / Neurogenic claudication
Motor_deficit	Present, stable / Present, progressive / blank (absent)
Sensory_loss	Yes / No
Reflex_change	Yes / No
Suspected_cauda_equina	Yes / No
Suspected_malignancy_weight_loss_or_night_pain	Yes / No
Suspected_infection_fever_IVDU_recent_procedure	Yes / No
Significant_trauma	Yes / No
Inflammatory_features_morning_stiffness	Yes / No
Conservative_treatment_at_least_6_weeks	Yes / No
Response_to_conservative_treatment	Responded / No response / Not given
Surgery_or_intervention_under_consideration	Yes / No
Previous_lumbar_MRI	Yes / No / Unknown
Previous_lumbar_surgery	Yes / No / Unknown

The models returned one row per request containing the request identifier and a single categorical judgement (Appropriate or Inappropriate); no rationale was requested or supplied. IVDU: intravenous drug use.

Supplementary Table S1. Sensitivity analysis excluding pre-imaging consideration of surgery or intervention from the rule-based instruments and from the derived rule (n = 147; reference outcome: management-changing MRI, 49/147).

Instrument	Se	Sp	PPV	NPV	Acc.	Youden	Scans, n
ACR-based rule, original	0.69	0.84	0.68	0.85	0.79	0.53	50
ACR-based rule, criterion excluded	0.41	0.90	0.67	0.75	0.73	0.31	30
NICE-based rule, original	0.67	0.92	0.80	0.85	0.84	0.59	41
NICE-based rule, criterion excluded	0.12	0.98	0.75	0.69	0.69	0.10	8
Four-item derived rule	0.84	0.81	0.68	0.91	0.82	0.64	60
Three-item derived rule, criterion excluded	0.55	0.85	0.64	0.79	0.75	0.40	42

Pre-imaging consideration of surgery or intervention denotes the treating clinician's judgement at the time of the request, based on the history and physical examination, that the patient might be a candidate for surgical or interventional treatment if imaging demonstrated a concordant structural lesion. 'Criterion excluded' rows are analytical constructs generated for this sensitivity analysis and do not represent alternative versions of the ACR or NICE recommendations; the excluded criterion corresponds to an analogous clinical concept in each instrument (ACR: candidacy for surgery or intervention; NICE: imaging expected to change management, operationalised here as pre-imaging consideration of surgery or intervention). After exclusion, the ACR-based rule then classifies imaging as appropriate only in the presence of a red flag or of symptoms persisting beyond six weeks despite at least six weeks of failed conservative treatment, and the NICE-based rule only in the presence of a red flag. The ACP-based rule and the large language model assessments are not shown, because the criterion cannot be removed from them mechanically. Four-item derived rule: red flag or neurological warning feature (motor deficit); neurogenic claudication; purely radicular pain; pre-imaging consideration of surgery/intervention. Three-item derived rule: the first three items only. ACR: American College of Radiology; NICE: National Institute for Health and Care Excellence; Se: sensitivity; Sp: specificity; Acc.: accuracy; PPV/NPV: positive/negative predictive value; Scans, n: number of examinations classified as indicated.

Supplementary Table S2. Individual characteristics of the seven patients with axial-predominant pain and a subsequent injection procedure whose management-changing MRI was not identified by the four-item rule.

No.	Age (y)	Sex	Symptom duration	Pain pattern	Conservative treatment ≥6 wk / response	Prior MRI	MRI finding	Subsequent intervention
1	24	F	<6 weeks	Axial only	Yes / responded	Yes	Disc herniation	Injection procedure
2	50	F	>12 weeks	Mixed axial–radicular	No / not given	Yes	Disc herniation	Injection procedure
3	47	M	>12 weeks	Axial only	No / responded	No	Degenerative change only	Injection procedure
4	60	M	<6 weeks	Axial only	No / not given	No	Degenerative change only	Injection procedure
5	48	F	>12 weeks	Axial only	No / responded	No	Degenerative change only	Injection procedure
6	58	F	6–12 weeks	Axial only	No / responded	No	Normal	Injection procedure
7	34	F	>12 weeks	Axial only	Yes / no response	Unknown	Degenerative change only	Injection procedure

None of the seven patients had a red flag or neurological warning feature, or pre-imaging consideration of surgery or intervention; six of the seven requests were classified as inappropriate by all five instruments (ACR, NICE, ACP, AI-1 and AI-2), and patient 7 was classified as appropriate by the ACR-based rule and AI-1 only. The eighth management-changing examination missed by the rule was in a patient with mixed axial–radicular pain of less than six weeks' duration who proceeded to surgery. MRI findings are shown for descriptive characterisation only and were not used in any appropriateness classification or in derivation of the rule. Conservative treatment column: whether at least six weeks of conservative treatment had been received before imaging / documented response to any conservative treatment. F: female; M: male; wk: weeks.

Supplementary Table S3. Run-to-run reproducibility of the large language model assessments (n = 147). Each model was run three times with the identical prompt, dataset and web interface: the primary assessment (August 2026, used in all main analyses) and two repeat runs in September 2026 in new, independent chat sessions.

Model	Comparison	Agreement, % (n/147)	Cohen's κ
AI-1 (Claude)	August vs September run 1	91.8 (135/147)	0.82
AI-1 (Claude)	August vs September run 2	91.8 (135/147)	0.82
AI-1 (Claude)	September run 1 vs run 2	94.6 (139/147)	0.88
AI-1 (Claude)	Identical decision in all three runs	89.1 (131/147)	–
AI-2 (ChatGPT)	August vs September run 1	91.8 (135/147)	0.83
AI-2 (ChatGPT)	August vs September run 2	91.2 (134/147)	0.82
AI-2 (ChatGPT)	September run 1 vs run 2	93.9 (138/147)	0.87
AI-2 (ChatGPT)	Identical decision in all three runs	88.4 (130/147)	–

Run	Inappropriate, n (%)	κ vs ACR	κ vs NICE	κ vs ACP	Se	Sp	PPV	NPV	Youden
AI-1, August (primary)	95 (64.6)	0.91	0.77	0.85	0.67	0.81	0.63	0.83	0.48
AI-1, September run 1	99 (67.3)	0.85	0.76	0.88	0.61	0.82	0.62	0.81	0.43
AI-1, September run 2	93 (63.3)	0.82	0.74	0.88	0.65	0.78	0.59	0.82	0.43
AI-2, August (primary)	85 (57.8)	0.77	0.69	0.77	0.73	0.73	0.58	0.85	0.47
AI-2, September run 1	83 (56.5)	0.74	0.67	0.80	0.76	0.72	0.58	0.86	0.48
AI-2, September run 2	92 (62.6)	0.87	0.79	0.87	0.69	0.79	0.62	0.84	0.48

Upper panel: agreement between runs. Lower panel: agreement of each run with the operationalised guideline rules and performance of each run in predicting a management-changing MRI (reference outcome: surgery or interventional procedure, 49/147). Differences between runs were confined to borderline presentations in which guideline logic is itself least explicit — chiefly chronic radicular or mixed pain without an adequate conservative treatment trial, short-duration pain unresponsive to conservative treatment, and requests in which surgery or intervention was under consideration despite a clinical response to conservative treatment. Model labels as displayed by the interfaces: AI-1 'claude-fable-5' (Anthropic), AI-2 'GPT-5.6 Sol' (OpenAI). Se: sensitivity; Sp: specificity; PPV/NPV: positive/negative predictive value.

Supplementary Table S4. Completed TRIPOD checklist for reporting of the derivation and internal assessment of the clinical decision rule (TRIPOD 2015, development items).

Item	Topic	Checklist item	Section
1	Title	Identify the study as developing a prediction model, target population and outcome	Title
2	Abstract	Summary of objectives, design, setting, participants, sample size, predictors, outcome, statistical analysis, results and conclusions	Abstract
3a	Background	Medical context, rationale for developing the model and reference to existing models	1
3b	Objectives	Specify the objectives, including whether the study describes development or validation	1 (final paragraph)
4a	Source of data	Describe the study design or source of data	2.1
4b	Source of data	Key study dates including start and end of accrual and follow-up	2.1 (April–July 2026)
5a	Participants	Key elements of the study setting (location, number of centres) and eligibility	2.1
5b	Participants	Eligibility criteria for participants	2.1

5c	Participants	Details of treatments received, if relevant	2.2 (conservative treatment)
6a	Outcome	Clearly define the outcome, including how and when assessed	2.5
6b	Outcome	Report any actions to blind assessment of the outcome	2.2, 2.4 (pre-imaging variables and LLM assessments blinded to outcome)
7a	Predictors	Clearly define all predictors, including how and when measured	2.2, 2.3
7b	Predictors	Report any actions to blind assessment of predictors for the outcome	2.2 (contemporaneous pre-imaging documentation)
8	Sample size	Explain how the study size was arrived at	2.1; 4 (Limitations)
9	Missing data	Describe how missing data were handled	2.2; 4 (prior imaging incomplete, not modelled)
10a	Statistical analysis	How predictors were handled in the analyses	2.6 (binary criteria)
10b	Statistical analysis	Type of model, model-building procedures and internal validation	2.6, 2.7
10d	Statistical analysis	Measures used to assess model performance and, if relevant, compare models	2.6, 2.7
11	Risk groups	Provide details on how risk groups were created, if done	Not applicable (binary rule)
13a	Participants	Flow of participants, number with and without the outcome	2.1; 3.1
13b	Participants	Characteristics of participants	3.1; Table 1
14a	Model development	Number of participants and outcome events in each analysis	3.1, 3.4
14b	Model development	Unadjusted association between each candidate predictor and outcome	3.4
15a	Model specification	Present the full prediction model	3.4; Figure 2A
15b	Model specification	Explain how to use the prediction model	3.4; Figure 2A
16	Model performance	Report performance measures (with confidence intervals) for the model	3.4; Table 4; Supplementary Table S1
18	Limitations	Discuss limitations (non-representative sample, few events, missing data, overfitting)	4 (Limitations)
19b	Interpretation	Give an overall interpretation of the results considering objectives, limitations and similar studies	4
20	Implications	Discuss the potential clinical use and implications for future research	4; 5
21	Supplementary information	Provide information about the availability of supplementary resources	Supplementary Materials; Data Availability Statement
22	Funding	Give the source of funding and the role of the funders	Funding statement

The study is a derivation study with internal assessment (stratified five-fold cross-validation of benchmark models and bootstrap confidence intervals); no external validation was performed. Items 10c, 10e, 12, 17 and 19a apply to validation studies only and are therefore not applicable.
